# Swept-Source Wide-Field OCT and OCTA (24 × 20 mm and 26 × 21 mm) in Inherited Retinal Dystrophies: First Clinical Experience with Two Novel Devices

**DOI:** 10.3390/jcm15156015

**Published:** 2026-08-02

**Authors:** Ghazaleh Farmand, Ulrich Kellner

**Affiliations:** 1 Rare Retinal Disease Center, AugenZentrum Siegburg, MVZ Augenärztliches Diagnostik- und Therapiecentrum Siegburg GmbH, 53721 Siegburg, Germany; g.farmand@osg.de; 2 RetinaScience, 53225 Bonn, Germany

**Keywords:** inherited retinal dystrophies, wide-field retinal imaging, optical coherence tomography, OCT angiography, ultra wide-field retinal imaging

## Abstract

**Background**: Optical coherence tomography (OCT) and OCT angiography (OCTA) retinal imaging in inherited retinal dystrophies (IRD) has been limited to the posterior pole and central midperiphery (up to about 16.5 × 16.5 mm). Two novel commercially available swept-source (SS) OCT/-OCTA devices provide the possibility of wide-field (WF) evaluation of retinal and choroidal structures, including the vasculature, in a single examination. **Methods**: A limited consecutive series of 16 IRD patients were examined with a BMizar (400 kHz, 24 × 20 mm scan width) and a Dream OCT (200 kHz, 26 × 21 mm scan width) in addition to the normal clinical examination protocol. This series included patients with retinitis pigmentosa, cone-rod dystrophy, macular dystrophy and autosomal recessive bestrophinopathy. In addition, 12 healthy probands were examined. **Results**: WF-SS-OCT/-OCTA enabled the detection of retinal, choroidal and choriocapillaris alterations in the macular and midperiphery in a short, single examination session of up to 15 s. Even small foveal lesions and a small silent macular neovascularization were detected on WF screening. Regional alterations of choroidal and choriocapillaris flow patterns were identified. These were mostly in correspondence with areas that appeared clinically affected, but unexpected lesions were identified as well. Occlusion of peripheral retinal vessels was seen in retinitis pigmentosa, though flow was detected in retinal vessels, which were difficult to distinguish on fundus images. In one patient with nystagmus, WF-SS-OCT/-OCTA was performed, whereas standard OCT volume scan could not be obtained. The most frequent artifact were horizontal lines of misalignment, which did not interfere with the detection of pathologies. **Conclusions**: Both WF-SS-OCT/-OCTA devices provide detailed insights in structural and vascular retinal and choroidal alterations in a single, short examination. Larger series of IRD patients examined with WF-SS-OCT/-OCTA promise to provide novel insights into the pathology of IRDs.

## 1. Introduction

For technical reasons, and due to the relevance of the macular area for visual function, retinal imaging has predominantly focused on the posterior pole and midperiphery, with an image field of 30 to 55 degrees (up to 16.5 mm horizontal width). Ultra-wide-field (UWF) imaging, including the far periphery, first became available for fundus photography, fundus autofluorescence and fluorescein angiography. Without montage, optical coherence tomography (OCT) was confined to the posterior pole and midperiphery (up to 16.5 mm horizontal width) and OCT angiography (OCTA) to even smaller areas (up to 12 × 12 mm). Recently, two novel swept-source devices (SS-OCT/OCTA) were introduced, extending the imaged area to 24 × 20 mm (BMizar) and 26 × 21 mm (Dream OCT), with one measurement providing an unprecedented non-invasive view on retinal and choroidal structures, including the vasculature [1,2,3]. So far, these SS-OCTA devices have mostly been used to analyze more peripheral vascular alterations in chorioretinal vascular disorders like diabetic retinopathy, retinal vein occlusion or Vogt–Koyanagi–Harada disease [4,5,6,7,8,9].

Unfortunately, the terminology used by distributors as well as in the literature is misleading [3]. While 12 × 12 mm OCTA scans have been marketed as wide-field (WF), the novel devices have been frequently termed UWF. The difficulties of area definition have been discussed in detail previously [3]. In adherence with this definition, we use the term WF-SS-OCT/-OCTA. UWF-SS-OCT/-OCTA with visualization peripheral to the vortex veins can only be achieved by montage with both novel devices.

The importance of WF/UWF imaging in inherited retinal dystrophies (IRD) was underlined several years ago [10], but only few studies have reported wider-field OCT/-OCTA in IRDs. Mid-peripheral alterations of the choroidal vasculature in retinitis pigmentosa have been described using 12 × 12 mm OCTA [11,12]. Using 24 × 20 mm WF-SS-OCTA, choroidal and retinal vascular alterations have been reported in one series of retinitis pigmentosa patients [13]. Peripheral loss of retinal vasculature in retinitis pigmentosa has been recently reported based on UWF fundus photography evaluation [14]. In a patient with choroideremia, choroidal vascular flow was reduced in 24 × 20 mm WF-SS-OCTA, and preservation of choroidal vessels was correlated with preserved areas of retinal pigment epithelium [15]. WF-SS-OCT/-OCTA has been used to examine peripheral retinoschisis in x-linked retinoschisis [16] as well as familial exudative vitreoretinopathy [17].

For the current study, we used this opportunity to examine IRD-patients with both novel WF-SS-OCT/-OCTA devices in parallel to test the clinical usefulness of this imaging approach as well as to identify potential differences between both devices. To the best of our knowledge, this is the first comparison between both devices using the widest single-scan imaging field; a previous comparison focused on 3 × 3 mm macular OCTAs [18].

## 2. Materials and Methods

For a short period of one week, two commercially available WF-SS-OCT/OCTA devices were directly compared in our clinic: TowardPI BMizar (TowardPI Medical Technology Group Co., Ltd., Beijing, China) and Intalight Dream OCT (Intalight Inc., San Jose, Ca, USA). Both devices are available in more than one configuration, which differ in the angle of view as well as the measurement frequency. For testing, a BMizar (A-scan rate 400 kHz, 1060 nm laser, maximal 24 × 20 mm scan width, axial resolution ≤6 µm, transverse resolution ≤10 µm, software version VS15)) and a Dream OCT (A-scan rate 200 kHz, 1030–1070 nm laser, maximal 26 × 21 mm scan width, axial resolution ≤5.5 µm, transverse resolution ≤15 µm, software version v1.0.403A) were used [1,2].

Both devices allowed retinal images either as volume or single scan. WF-SS-OCT volume scans resulted in up to 1250 single horizontal scans, which can be better visualized in different retinal and choroidal layers in enface mode. WF-SS-OCTA images were separated in several retinal and choroidal layers; for the present evaluation, choroidal vascular flow, choriocapillaris flow, avascular layer flow and superficial retinal vascular flow were selected. Both devices provide multiple features for detailed adjustments of the measurement and evaluation protocols. Due to the limited time, only the predefined imaging protocols were used.

A consecutive series of IRD patients undergoing first or follow-up clinical evaluation were asked to consent to be examined with both devices; none of them declined. In total, 16 patients were examined (macular dystrophies: 6, retinitis pigmentosa 6, cone-rod-dystrophy: 2, autosomal recessive bestrophinopathy: 1, congenital stationary night blindness: 1). All patients underwent the normal clinical protocol of refraction and visual acuity testing, visual field examination, biomicroscopy of the anterior segment, and ophthalmoscopy, as well as the normal imaging protocol of wide-angle fundus photography (133 degrees, about 19 × 19 mm; Clarus 700, Carl Zeiss, Jena, Germany), 30° (about 9 × 9 mm) and 55° (about 16.5 × 16.5 mm) degree fundus and near-infrared autofluorescence (FAF and NIA, HRA, Heidelberg Engineering, Heidelberg, Germany), 30° and 55° degree Spectral Domain OCT, and 6 × 6 mm OCTA (Spectralis, Heidelberg Engineering, Heidelberg, Germany) [19]. In addition, 12 healthy volunteers agreed to be examined. All images were obtained by trained photographers. All examinations were performed following informed consent in accordance with the Declaration of Helsinki and in agreement with the use of CE-certified imaging devices for research as approved by the local ethics committee.

In this small study, evaluation of all images was done unmasked and based on subjective comparison. A masked comparison was not possible due to the difference in imaged areas obvious to any grader.

## 3. Results

### 3.1. Normal Retinal and Choroidal Vasculature

Image acquisition time to obtain WF-SS-OCT/-OCTA was short with both devices (about 10–15 s). Due to the difference in A-Scan rate between both devices, we did not perform a detailed comparison of examination times. In addition, examination time depends on the imaging protocols due to different optic solutions in both devices: whereas BMizar uses one lens, different lenses need to be adjusted for measurement of posterior pole, WF- or anterior segment imaging for Dream OCT. With both devices, examination time for a single combined WF-SS-OCT/-OCTA measurement was markedly shorter compared to standard subsequent measurements of wide-field OCT volume scan and posterior pole 6 × 6 mm OCTA. One patient with marked nystagmus was examined. In this patient, only measurement with Dream OCT obtained reliable images, while only single OCT scans were possible with BMizar and Spectralis OCT.

**Figure 1:**
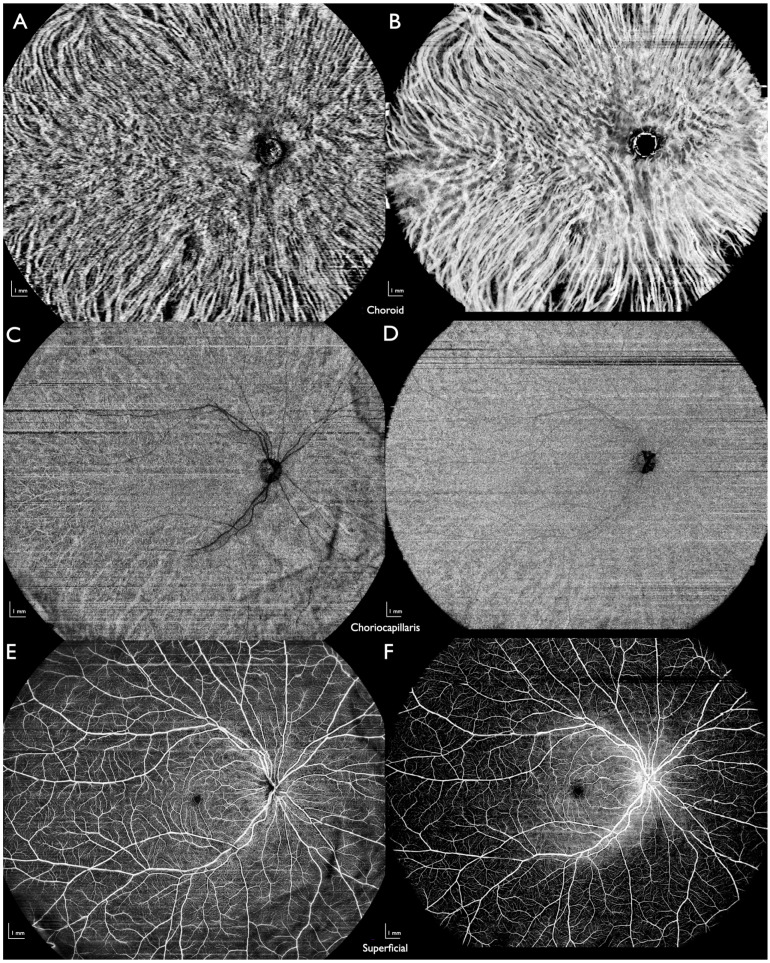
Normal distribution of vascular flow patterns in the choroid ((**A**) BMizar 24 × 20 mm, (**B**) Dream OCT, 26 × 21 mm), choriocapillaris ((**C**) BMizar, (**D**) Dream OCT) and superficial retinal vessels ((**E**) BMizar, (**F**) Dream OCT) in a 46-year-old female volunteer. Large choroidal vessels are more clearly defined by Dream OCT (**B**); more details of the choriocapillaris are visible with BMizar (**C**). Contrast between retinal vessels and background is higher with Dream OCT (**F**) but more homogeneous with BMizar.

In normal probands, wide-field retinal and choroidal flow patterns were defined with both devices (Figure 1). There was no marked difference between both devices. Subjectively, choroidal vasculature flow was more focused with the Dream OCT, whereas choriocapillaris flow showed more details with the BMizar. Retinal vascular flow showed a more homogeneous background with BMizar, while more smaller vessels were visible at the posterior pole compared to the periphery with Dream OCT. Despite the high frequency of measurements, horizontal lines with artifacts were visible on nearly all images in all probands and patients; these lines did not interfere with the identification of pathologies in the present series. In the IRD-patient with congenital stationary night blindness, WF-SS-OCTA retinal and choroidal flow patterns were normal.

### 3.2. IRD Patients

#### 3.2.1. Retinitis Pigmentosa

**Figure 2:**
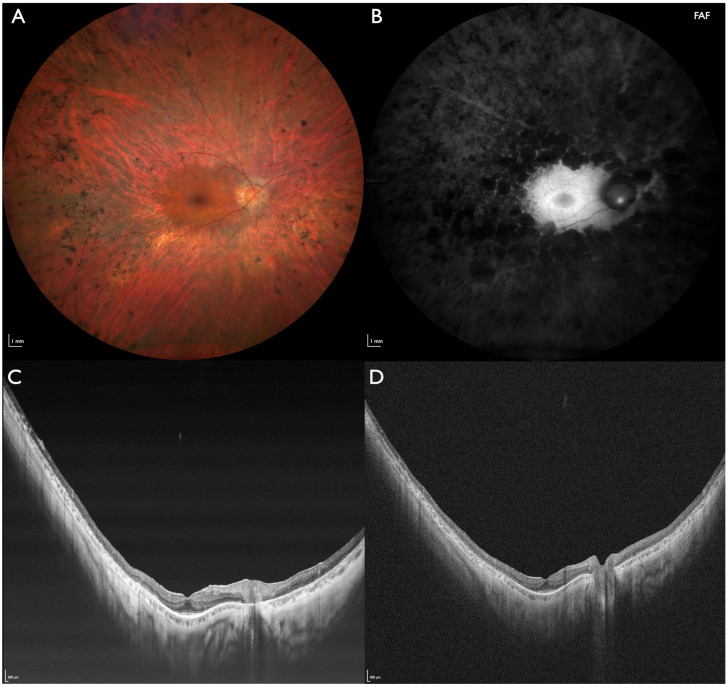
In a 55-year-old male patient with *PRPF31*-associated autosomal dominant retinitis pigmentosa, fundus photography ((**A**) Clarus 19 × 19 mm) demonstrated attenuated vessels, midperipheral and peripheral retinal pigment epithelium atrophy as well as multiple pigmented flecks. Fundus autofluorescence ((**B**) Clarus) showed midperipheral and peripheral markedly reduced FAF intensity and preserved intensity at the posterior pole with a pericentral ring of increased intensity. WF-SS-OCT ((**C**) BMizar horizontal length 24 mm, (**D**) Dream OCT horizontal length 26 mm) showed a small foveal defect in the ellipsoid zone and midperipheral and peripheral loss of photoreceptor-associated layers as well as a mild epiretinal membrane. More details are visible in the BMizar scan (**C**).

WF-SS-OCT provided structural information of foveal and peripheral retinal layers in eyes with retinitis pigmentosa. Small foveal alterations were detectable, as well as peripheral loss of photoreceptors. Subjectively, BMizar single scans appeared less granular and showed more details compared to Dream OCT (Figure 2C,D).

**Figure 3:**
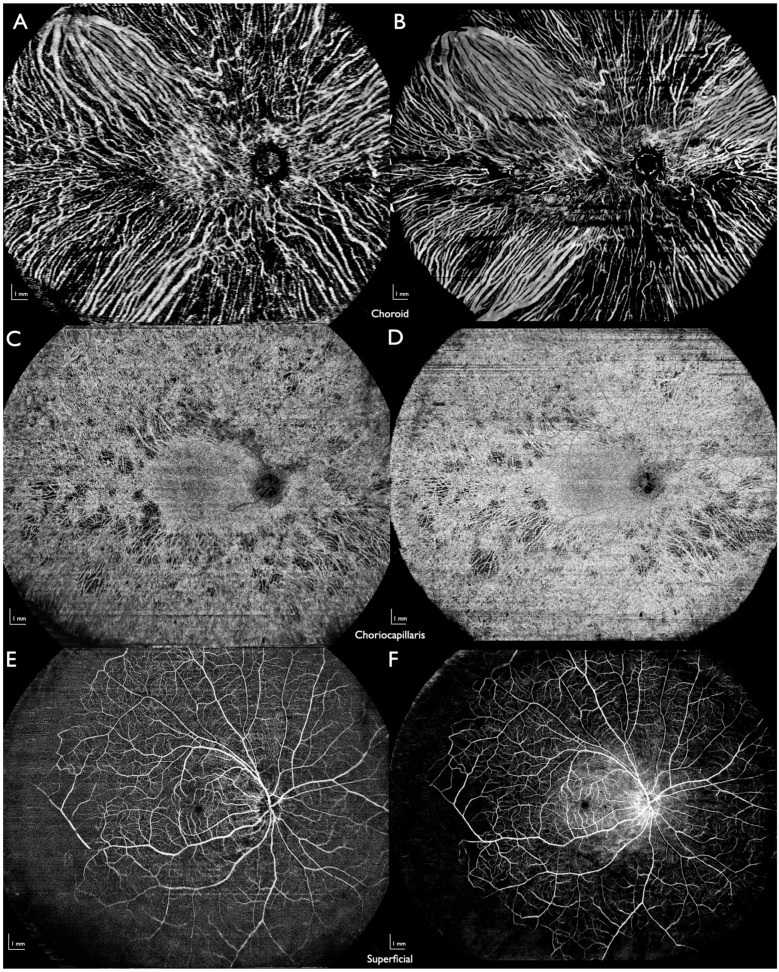
Same eye as in Figure 2: Vascular flow patterns in the choroid ((**A**) BMizar 24 × 20 mm, (**B**) Dream OCT 26 × 21 mm) showed reduced density of vessels. Midperipheral and peripheral irregularities of choriocapillaris flow ((**C**) BMizar, (**D**) Dream OCT) was more detailed in BMizar (**C**). Peripheral absence of flow in superficial retinal vessels ((**E**) BMizar, (**F**) Dream OCT) was obvious in both techniques, while pericentral vessels could be better observed with BMizar (**E**) and peripheral vessel with Dream OCT (**F**). Flow was present in midperipheral and peripheral retinal vessels that were difficult to visualize on fundus photography (Figure 2A).

**Figure 4:**
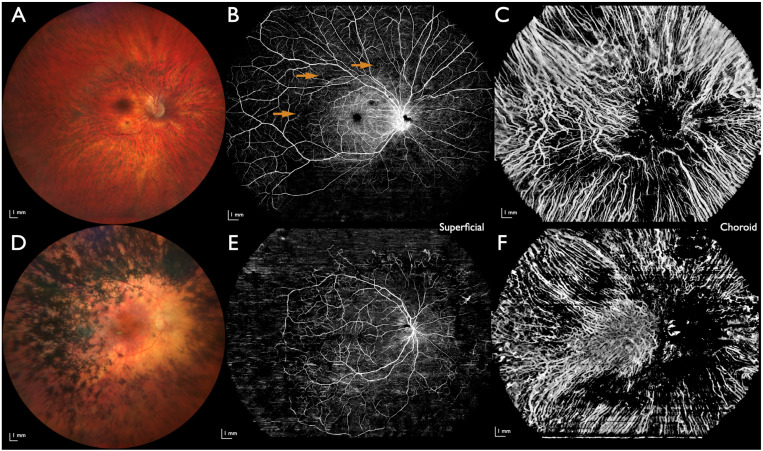
Fundus photography (Clarus, 19 × 19 mm), retinal vessel and choroidal vessel flow obtained with Dream OCT (26 × 21 mm) in two patients with retinitis pigmentosa. (**A**–**C**) A 29-year-old male patient with *TTLL5*-associated autosomal recessive sectorial retinitis pigmentosa. Retinal flow was markedly reduced below the lower temporal vascular arcade and slightly reduced in the rod-dominated region temporal of the fovea as well as superior to the upper temporal vascular arcade (arrows). Choroidal vessel flow was reduced in the same areas. (**D**–**F**) A 66-year-old female patient with simplex retinitis pigmentosa and inconclusive molecular genetic findings. Retinal vascular flow was absent in the periphery, but more vessels were detectable compared to the fundus image. Choroidal vessel flow was predominantly reduced in the midperiphery.

WF-SS-OCTA showed multiple alterations in different layers (Figure 3 and Figure 4). Choroidal vasculature showed a reduced vessel density compared to normal eyes (Figure 1) or eyes with macular dystrophy. In the choriocapillaris midperipheral and peripheral, patchy areas of reduced or absent flow were detectable. This was more severe in the midperiphery and appeared to be better visualized by BMizar (Figure 3). On ophthalmoscopy, retinal vessels are attenuated in most patients with retinitis pigmentosa (Figure 2A and Figure 4). Peripheral loss of retinal flow was demonstrated in both sectorial retinitis pigmentosa, as well as circumferential retinitis pigmentosa. It is interesting that retinal vascular flow was demonstrated in small peripheral vessels that were difficult to see on ophthalmoscopy or fundus images.

#### 3.2.2. Cone-Rod Dystrophy

**Figure 5:**
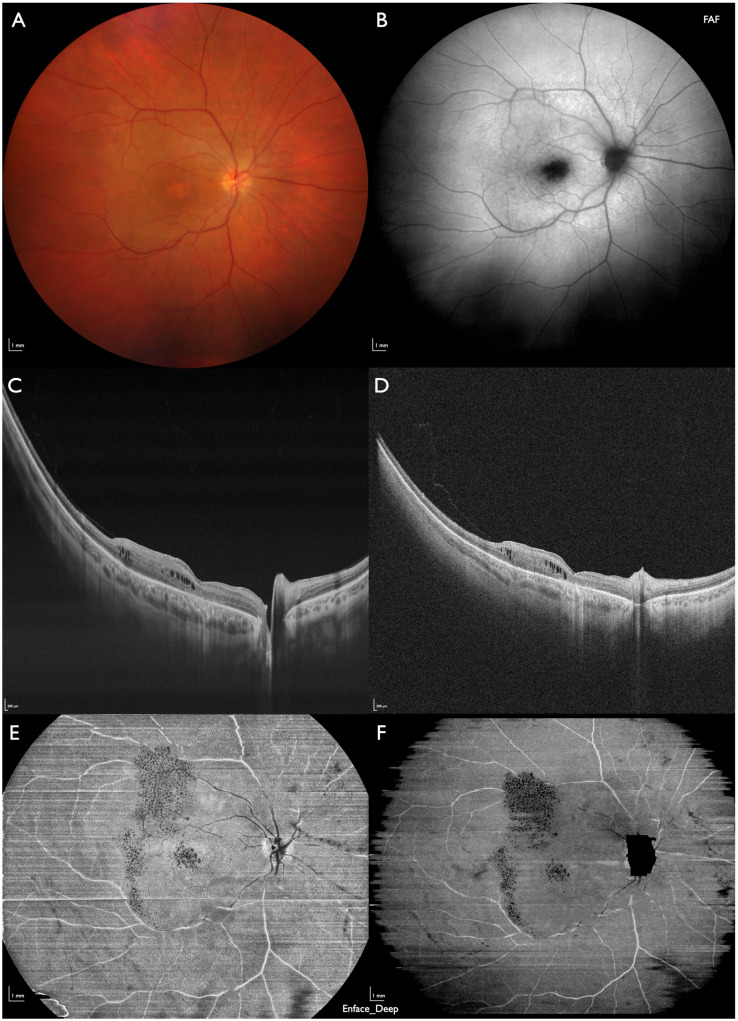
In a 44-year-old female patient with autosomal dominant cone–rod dystrophy and inconclusive molecular genetic findings, fundus photography ((**A**) Clarus 19 × 19 mm) demonstrated a central atrophic lesion. Fundus autofluorescence ((**B**) Clarus) showed central markedly reduced FAF intensity surrounded by an area of fleck-like reduced or increased FAF intensity extending beyond the temporal vascular arcades and nasal of the optic disk. WF-SS-OCT ((**C**) BMizar, (**D**) Dream OCT) showed irregular foveal retinal layers and retinal pigment epithelial atrophy, as well as pericentral intraretinal cystoid spaces. More details are visible in the BMizar scan (**C**). The enface images ((**E**) BMizar 24 × 20 mm, (**F**) Dream OCT 26 × 21 mm) demonstrate the distribution of central and midperipheral intraretinal cystoid lesions.

WF-SS-OCT provided structural information of foveal and peripheral retinal layers in eyes with cone–rod dystrophy (Figure 5). Foveal alterations, including retinal pigment epithelial atrophy, were detectable, as well as pericentral cystoid intraretinal spaces in both vertical and enface images. Subjectively, BMizar images appeared less granular and showed more details compared to Dream OCT (Figure 5C,D).

**Figure 6:**
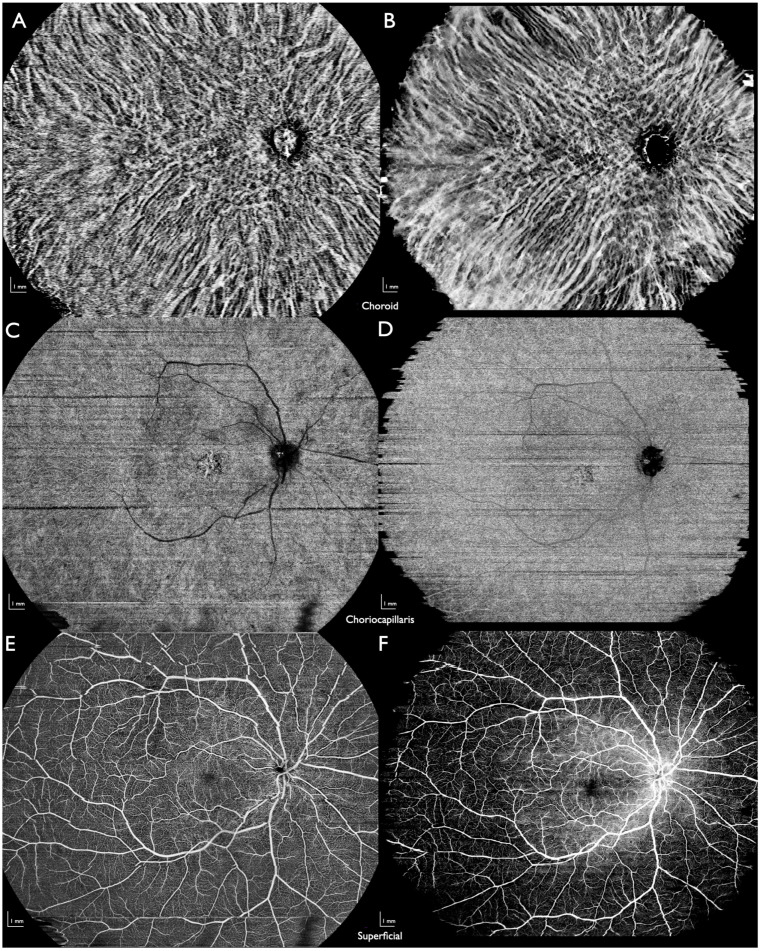
Same eye as Figure 5: Vascular flow patterns in the choroid ((**A**) BMizar 24 × 20 mm, (**B**) Dream OCT 26 × 21 mm) showed potentially reduced density of vessels in the foveal area, while midperipheral and peripheral flow appeared normal. Central irregularities of choriocapillaris flow ((**C**) BMizar, (**D**) Dream OCT) were more detailed in BMizar (**C**). Midperipheral areas with reduced intensity correspond to areas with intraretinal cystoid spaces in this patient (Figure 5C,D). Superficial retinal vessel flow appeared normal ((**E**) BMizar, (**F**) Dream OCT).

WF-SS-OCTA showed multiple alterations in different layers (Figure 6). Choroidal vascular density was slightly reduced in the foveal area (Figure 6A,B). In the choriocapillaris, irregular subfoveal flow was better visualized with BMizar. The retinal vasculature appeared normal; however, in contrast to retinitis pigmentosa, no eyes with markedly progressed phenotypes could be examined.

#### 3.2.3. Autosomal Recessive Bestrophinopathy (ARB)

**Figure 7:**
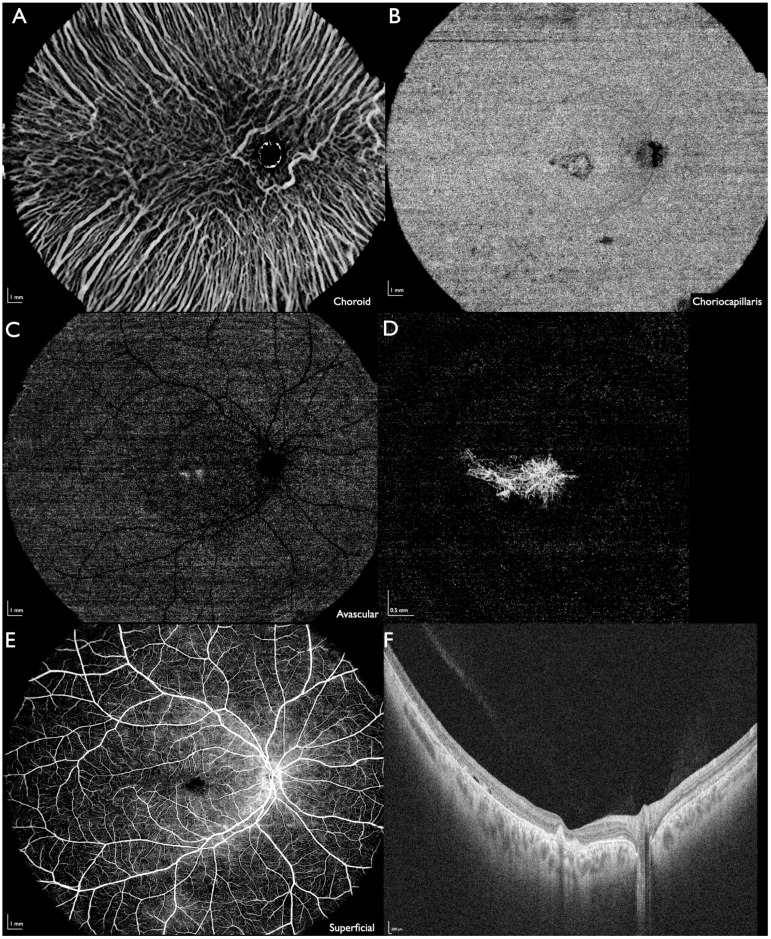
In a 30-year-old female patient with *BEST1*-associated autosomal recessive bestrophinopathy (Dream OCT 26 × 21 mm), choroidal flow appeared moderately reduced at the posterior pole (**A**). Choriocapillaris flow was irregular at the posterior pole (**B**). A presence of flow within the avascular zone was detected by wide-field imaging (**C**); a smaller 6 × 6 mm central scan identified a silent macular neovascularization (MNV; (**D**)). Retinal vascular flow appeared normal (**E**). Structural OCT showed subfoveal material corresponding to the silent MNV and outer retinal layer irregularities temporal to the fovea, as well as milder irregularities nasal of the optic disc (**F**).

WF-SS-OCT detected foveal and mid-peripheral alterations in the outer retinal layers in one ARB patient (Figure 7F). WF-SS-OCTA showed moderately reduced choroidal vasculature flow at the posterior pole and subfoveal irregularities in the choriocapillaris. A silent macular neovascularization was detected in the avascular layer as increased flow with both BMizar (not shown) and Dream OCT (Figure 7C), but for details, a smaller scan area was required (Figure 7D). Retinal vasculature appeared normal.

#### 3.2.4. Macular Dystrophy

**Figure 8:**
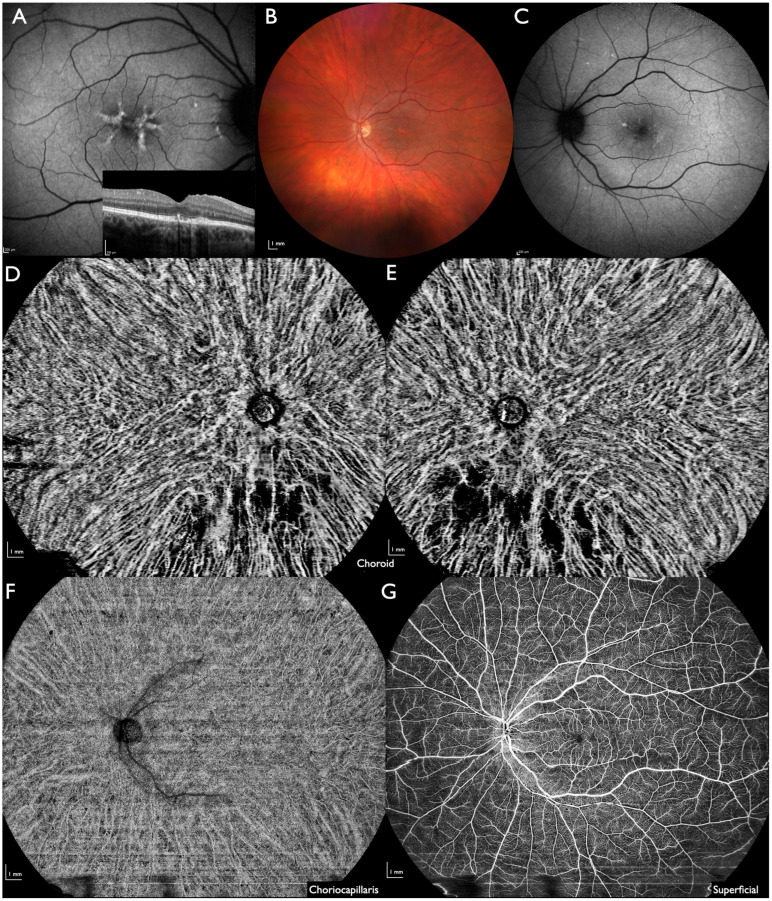
In a 66-year-old male patient with macular dystrophy, fundus autofluorescence (FAF) of the right eye showed pattern-like lines of increased FAF intensity ((**A**) Spectralis, 9 × 9 mm), associated with subretinal material on OCT (inset). On the left eye, fundus photography showed foveal irregularities as well as a mid-peripheral area of lighter appearance below the lower vascular arcade ((**B**) Clarus 19 × 19 mm). FAF showed fleck-like increased or reduced FAF intensity at the posterior pole and few fleck-like lesions beyond the vascular arcades, but no alterations in the mid-peripheral area seen on fundus photography ((**C**) Spectralis, 16.5 × 16.5 mm). WF-SS-OCTA (BMizar 24 × 20 mm) showed bilateral midperipheral regional absence of the choroidal flow on the right (**D**) and left eye (**E**), corresponding to the atrophic area seen in (**B**). In contrast, choriocapillaris (**F**) and retinal vessel flow (**G**) appeared normal.

In patients with macular dystrophies, WF-SS-OCT revealed structural abnormalities at the posterior pole similar to patients with cone–rod dystrophies. On WF-SS-OCTA, subfoveal irregularities were seen (Figure 8F), whereas retinal vasculature appeared normal (Figure 8G). In one patient, bilateral midperipheral areas of absent flow were present in the choroidal vasculature. This area corresponded to a different coloration on fundus photography, but no signs of atrophy appeared on fundus autofluorescence or staphyloma on WF-SS-OCT (Figure 8B–E).

## 4. Discussion

This study presents the first series of IRD patients examined with two novel WF-SS-OCT/-OCTA devices, demonstrating peripheral alterations of the retinal vasculature as well as alterations of the choriocapillaris and the choroidal vasculature in IRDs.

Although the number of patients examined was small, this study underlines the importance of wide-field OCT and OCTA imaging when examining IRD patients. The examination time is short and provides WF-SS-OCT/-OCTA information with just one measurement, whereas two separate tests would be needed with other devices. A short examination time is valuable in patients with reduced visual function and problems with stable fixation. The WF-SS-OCT/-OCTA results can be evaluated and displayed in multiple different enface layers; only few could be shown in this manuscript.

Multiple pathologies were identified; some of them were not identifiable with other devices. The present findings confirm previous reports of peripheral retinal vessel occlusion in retinitis pigmentosa [13,14]. Comparison of fundus images and WF-SS-OCTA indicate that retinal vascular flow can be detected in retinal vessels, which are difficult to detect on fundus photography; therefore, WF-SS-OCTA could provide an advantage in determining occlusion of peripheral retinal vessels in IRD. Some retinitis pigmentosa patients might show peripheral leakage on fluorescein angiography [20], which cannot be detected on OCTA. Clinical examination must guide the decision, whether UWF fluorescein angiography is indicated in addition to WF-SS-OCTA. Notably, occlusion of peripheral retinal vessels in retinitis pigmentosa can be seen in the same publication as well, though it was not discussed by the authors [20].

In addition, WF-SS-OCTA showed regional differences in choriocapillaris and choroidal flow in various IRDs, nearly always corresponding with the retinal pathology especially in sectorial IRD phenotypes. In retinitis pigmentosa, similar choroidal and choriocapillaris vascular alterations have been previously reported at the posterior pole using 6 × 6 to 12 × 12 mm OCTA [11,12,21,22] and in the midperiphery using 24 × 20 mm WF-SS-OCTA [13]. In cone–rod and macular dystrophies, subfoveal choriocapillaris flow was reduced, as has been observed in macular OCTA previously [23]. In one patient with macular pattern dystrophy and few fleck-like midperipheral lesions, bilateral sectorial mid-peripheral loss of choroidal vascular flow was detected (Figure 8). In a single case, this could be either coincidental or associated with macular dystrophy. Larger series of IRD patients need to be examined with WF-SS-OCTA to define whether more unexpected pathologic alterations are present than can be detected with standard field examinations.

It is important that WF-SS-OCT provided detailed information on macular and foveal pathologies in one single examination (Figure 2 and Figure 7). WF-SS-OCT therefore appears to be a useful screening technique that allows us to focus on detected pathologies with subsequent scans of higher resolution. Similarly, WF-SS-OCTA detected bilateral silent macular neovascularization based on presence of flow in the avascular layer; a smaller higher resolution scan confirmed this finding in detail (Figure 7).

There were limited subjective differences between both devices when used with the settings predefined by the distributors in this first clinical experience with both devices. Subjective slight advantages of BMizar included more details of macular pathology in single line scans, slightly better enface differentiation, more details in the choriocapillaris vascular flow, and a more homogeneous view of retinal vasculature flow. Subjective slight advantages of Dream OCT included the more detailed separation of choroidal vascular flow and the better detection and differentiation of macular neovascularization. Due to the limited time, we were unable to test adjustments to the predefined settings, e.g., denoise functions or different tracking modes, which most likely would improve the image quality for both current devices. This could also explain the different results in the patient with nystagmus. Further studies with multiple patients with nystagmus would be needed to establish a definite difference. In addition, software upgrades have been announced for both devices, which will improve image quality.

Artifacts in the form of a few horizontal lines of discrete misalignment were present in nearly all images with both devices. These lines of misalignment affected all layers within the same measurement. These lines of misalignment did not interfere with the detection of retinal or vascular pathologies in the present series. A variation of tracking modes might be suitable to reduce or eliminate these artifacts. Further studies are needed to determine which imaging technique and protocol are most suitable for patients with limited vision.

In conclusion, both WF-SS-OCT/-OCTA devices are suitable for a fast examination of retinal and choroidal structure and vascularization in IRD patients, even in patients with limited cooperation. It can be expected that the examination of larger series of IRD patients will provide novel insights into choroidal and retinal pathology of different IRDs. Potentially, comparison of retinal and choroidal vascular patterns using WF-SS-OCTA allows us to understand differences between humans and mammalian species used in animal experiments for ophthalmic research [24]. Both devices offer animal ocular imaging technology. Finally, it must be kept in mind that these WF-SS-OCT/-OCTA scans do not examine the entire fundus, and even more peripheral alterations might remain undetected [25].

## Data Availability

The data presented in this study are available on request from the corresponding author due to legal restrictions.
